# Reduced Performance During a Sentence Repetition Task by Continuous Theta-Burst Magnetic Stimulation of the Pre-supplementary Motor Area

**DOI:** 10.3389/fnins.2018.00361

**Published:** 2018-05-29

**Authors:** Susanne Dietrich, Ingo Hertrich, Florian Müller-Dahlhaus, Hermann Ackermann, Paolo Belardinelli, Debora Desideri, Verena C. Seibold, Ulf Ziemann

**Affiliations:** ^1^Department of Neurology & Stroke, Hertie Institute for Clinical Brain Research, University of Tübingen, Tübingen, Germany; ^2^Department of Psychology, Evolutionary Cognition, University of Tübingen, Tübingen, Germany; ^3^Department of Psychiatry and Psychotherapy, University Medical Center of the Johannes Gutenberg University, University of Mainz, Mainz, Germany

**Keywords:** cognitive control, inhibition, prediction, speech perception, top-down processing

## Abstract

The pre-supplementary motor area (pre-SMA) is engaged in speech comprehension under difficult circumstances such as poor acoustic signal quality or time-critical conditions. Previous studies found that left pre-SMA is activated when subjects listen to accelerated speech. Here, the functional role of pre-SMA was tested for accelerated speech comprehension by inducing a transient “virtual lesion” using continuous theta-burst stimulation (cTBS). Participants were tested (1) prior to (pre-baseline), (2) 10 min after (test condition for the cTBS effect), and (3) 60 min after stimulation (post-baseline) using a sentence repetition task (formant-synthesized at rates of 8, 10, 12, 14, and 16 syllables/s). Speech comprehension was quantified by the percentage of correctly reproduced speech material. For high speech rates, subjects showed decreased performance after cTBS of pre-SMA. Regarding the error pattern, the number of incorrect words without any semantic or phonological similarity to the target context increased, while related words decreased. Thus, the transient impairment of pre-SMA seems to affect its inhibitory function that normally eliminates erroneous speech material prior to speaking or, in case of perception, prior to encoding into a semantically/pragmatically meaningful message.

## Introduction

The supplementary motor area (SMA) can be subdivided into SMA proper and a more anterior part, i.e., the pre-SMA (Picard and Stick, 2001; Nachev et al., 2008). SMA proper—the border of which to pre-SMA is defined by the vertical crossing of the anterior commissure (Picard and Stick, 2001; Kim et al., 2010)—seems to be primarily involved in motor control tasks as an interface for movement initiation and temporal triggering in case of, e.g., syllable repetitions (Brendel et al., 2010). By contrast, the pre-SMA is assumed to be associated with cognitive control functions beyond the motor domain (Chouinard and Paus, 2010). For instance, pre-SMA was found to be involved in task switching (Kennerley et al., 2004), managing inhibitory mechanisms, e.g., in stop-signal tasks (Chen et al., 2009; Kwon and Kwon, 2013), response selection processes (Tremblay and Gracco, 2006, 2010), and complex sequencing, e.g., coordination of memory representations and temporal event structure (Kotz and Schwartze, 2010). Pre-SMA and SMA proper have, so far, not been considered to be part of the language system, but seem to play an important role during speech processing (Geranmayeh et al., 2017). As concerns cortico-cortical connectivity, pre-SMA is linked to regions in prefrontal cortex, inferior frontal cortex, angular gyrus, and ACC (Kim et al., 2010). Both, pre-SMA as well as the ACC are connected to Broca's region via the frontal aslant tract (Lawes et al., 2008; Oishi et al., 2008; Ford et al., 2010) which was found to be lateralized to the language-dominant hemisphere (Catani et al., 2012). Further, SMA shows a wide range of white matter connections with motor, language areas as well as the limbic system (Vergani et al., 2014). Thereby, parallel fiber bundles feed from SMA and ACC into inferior frontal regions and both were found to contribute to initiation of vocalization (Penfield and Welch, 1951; Simonyan and Horwitz, 2011). Lima et al. (2016) argued for the engagement of SMA across a variety of sound categories such as speech, nonspeech vocalizations, and music. Even the processing of socio-emotional information could be supported by anatomical connections between SMA and the limbic system (Rodigari and Oliveri, 2014). Both, left SMA and IFG (sub-) modules, are suggested to build a network (SMA-IFG-complex) relevant to speech reconstruction (Hertrich et al., 2016).

Regarding speech and language processing, pre-SMA was found to be relevant especially under conditions with high task demands, e.g., under time-critical circumstances. A recent functional magnetic resonance imaging (fMRI) study suggested that the “bottleneck” for understanding accelerated speech is limited by frontal cortex functions rather than auditory processing, as indicated by activation of pre-SMA and inferior frontal gyrus (IFG) when speech rate reaches the limits of intelligibility (Vagharchakian et al., 2012). Accordingly, a further fMRI study found increased left pre-SMA activation in individuals trained to comprehend ultra-fast speech at rates of 16–18 syllables per second (syl/s) (Dietrich et al., 2013a). The authors thereby suggested that pre-SMA is involved in the coordination of phonological-phonetic representations (left hemisphere) with syllable-prosodic event timing (right hemisphere), adjusting inner speech components to the timing of the incoming speech signal during listening (Dietrich et al., 2013a; Hertrich et al., 2013). In other words, pre-SMA has been suggested to trigger the mechanisms of predicting the next incoming speech items or reconstructing unintelligible linguistic items (Hertrich et al., 2016). Thus, reconstruction of missing information should be compatible to the already understood information, i.e., it should fit into the semantic and phonological context. Previous studies describing the role of pre-SMA during stop-signal tasks (Chen et al., 2009; Kwon and Kwon, 2013) and response selection processes (Tremblay and Gracco, 2006, 2010) suggested inhibitory mechanisms of the pre-SMA for managing these top-down aspects. This means that implausible top-down derived materials are inhibited whereas semantically plausible items are facilitated. Thus, pre-SMA is considered as a superordinate control structure with regard to inhibitory mechanisms on top-down processing during speech perception.

In order to test this hypothesized functional role of pre-SMA, continuous theta-burst transcranial magnetic stimulation (cTBS) was applied to healthy subjects. Selecting such kind of TMS protocol was found to induce inhibitory effects (virtual lesion) shortly (ca. 10 min) after stimulation for ~30 min (Huang et al., 2005; Murakami et al., 2015). For the present study, a pre/post1/post2 design was used predicting the TMS effect in the post1 compared to the two baseline measurements (pre/post2). The post-stimulation baseline was introduced in order to control for potential training effects during the experiment. The exact stimulation site was determined on the basis of previous fMRI data showing increased activity after training of ultra-fast speech comprehension (18 syl/s) (Dietrich et al., 2013a). Thus, pre-SMA was found to be a region relevant to accelerated speech comprehension. As a control stimulation site, left middle occipital gyrus (MOG) was chosen. MOG is involved in visual processing, but cannot be considered part of the auditory speech processing network (Restle et al., 2012; Murakami et al., 2015). Speech comprehension was measured with a sentence repetition task comprising sentences at distinct syllable rates ranging from moderately fast (8 syl/s) to ultra-fast speech (16 syl/s). The latter syllable rate was almost unintelligible to untrained listeners. We hypothesized that TMS-induced suppression of pre-SMA activity transiently reduces speech comprehension at high speech rates, due to an impairment of the reconstruction of unintelligible information by top-down processing. Furthermore, the assumption of an inhibitory function of pre-SMA was addressed by a qualitative analysis of errors in subjects' repetitions indicating unsuccessful attempts to reconstruct missing material. As a second hypothesis, suppression of pre-SMA was expected to impair the inhibition of implausible errors. Plausibility of reconstructed alternatives was considered in terms of semantic and/or phonological similarity between the reproduced material and the correct target words.

## Materials and methods

### Participants

Thirty-six adult volunteers participated in the study. Half of them (*n* = 18) underwent cTBS over left pre-supplementary motor area (pre-SMA, experimental group, mean age = 30.4, *SD* = 9.04), half of them served as a control group (*n* = 18) performing the repetition task with cTBS over left middle occipital gyrus (MOG, age = 29.2, *SD* = 11.18). All participants were male, right-handed (Edinburgh handedness inventory, laterality index of the experimental group: 88.9, *SD* = 13.23; control group: 83.5, *SD* = 16.56), native German speakers. None of them had any signs of neurological or psychiatric disorders, and all had normal hearing thresholds between −10 and +15 dB on each ear, tested for frequencies between 250 Hz and 4 kHz. Women were excluded from participation because the menstrual cycle can alter neuronal network excitability (Smith et al., 2002). All subjects provided written informed consent prior to participation, and the experimental procedures were approved by the ethics committee of the Medical Faculty of the University of Tübingen.

### Experimental design and procedure

**Figure 1:**
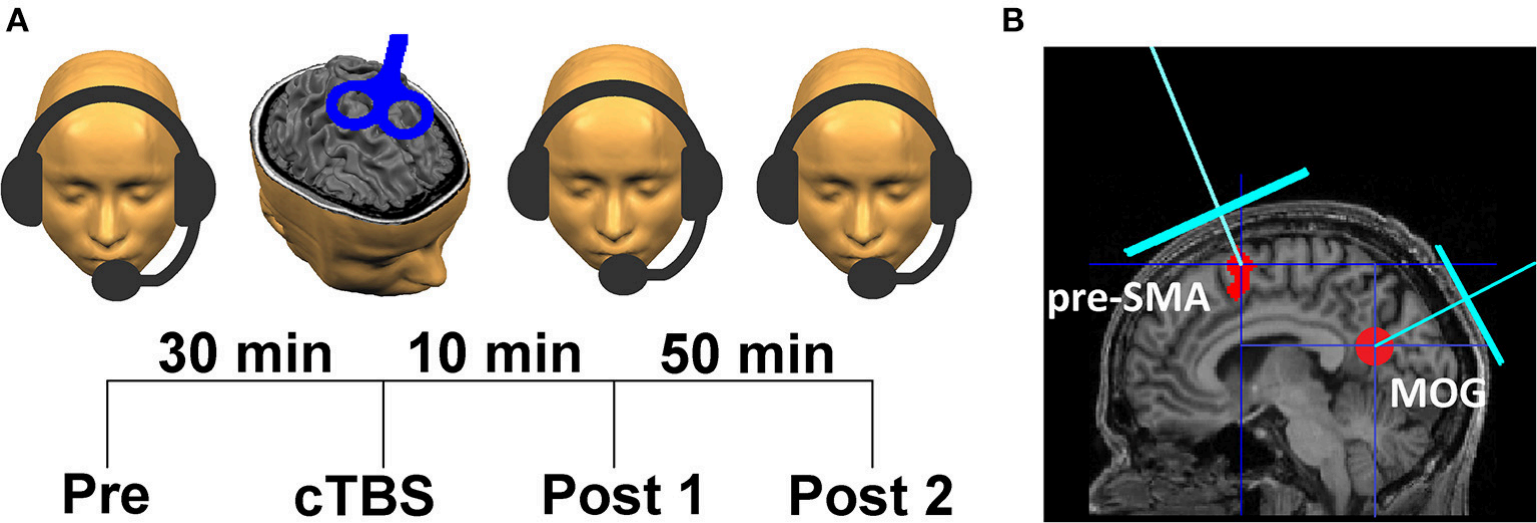
Experimental design. **(A)** Time course of an experimental session comprising three runs of intelligibility testing: pre baseline (pre), the test run (post1), and a post baseline (post2), **(B)** stimulation sites left pre-SMA (MNI coordinates x = −6, y = 9, z = 60) and left MOG (MNI coordinates x = −44, y = −74, z = 6) determined by neuro-navigation.

Participants performed sentence repetition tasks encompassing 135 sentences of a length of 18 syllables each (~10 words in order to limit memory load). The stimuli were based on newspaper materials and converted to speech by formant synthesis [“eloquence” implemented in the screen-reader software JAWS (freedom scientific, USA)] at five distinct speech rates: 8, 10, 12, 14, and 16 syl/s. Materials comprised three subtests for (pre-/post-) baseline and test measurements, with nine items per speech rate within each subtest (45 sentences per subtest, see Supplementary Table 1). The sentences were different at each recording point, and speech rates were randomized. The sentences were played via headphones in a sound attenuated room. Subjects were asked to repeat them “as accurately as possible” and “as fast as possible” after sentence offset, even when they failed to grasp all words. The subjects' repetitions were digitally recorded (M-audio Microtrack 2496, 16 bit, 44,100 samples/s) and underwent subsequent quantitative and qualitative evaluation of speech comprehension. Participants performed the repetition task prior to cTBS (pre), 10 min after cTBS (within the assumed time interval of the maximum virtual lesion effect, post1), and 60 min after cTBS (post2) (Figure 1A). The TMS protocol was adopted from Huang et al. (2005) who documented the return of changes in MEP amplitude to baseline levels after 60 min. Data from this group were from six subjects showing suppression at 25 and 45 min but no effect at 61 and 65 min (Huang et al., 2005). Since “training” effects from pre to post1 and post2 could occur when subjects adapt listening to synthetic speech, the post2-baseline was added in order to eliminate interference between “training” (increase of performance) and TMS inhibition (decrease of performance). Prior to the experimental session, a set of 18 practice trials was presented to the subjects to get acquainted with the test situation and the sound of the speech synthesizer. The three subsets of stimuli were rotated across participants with regard to the baseline (pre, post2) and test (post1) runs. The time intervals between pre, post1, and post2 testing were equal for the two subject groups (cTBS over pre-SMA or MOG). The repetition task per time point of measurement (pre or post1 or post2) had a duration of ~10 min.

### Transcranial magnetic stimulation

Prior to the main experiment, all participants were MRI-scanned to obtain a high-resolution T_1_-weighted anatomical dataset using a 3T Prisma Siemens scanner (resolution = 1 × 1 × 1 mm^3^) for the purpose of neuro-navigation. The pre-SMA stimulation site (MNI group coordinates x = −6, y = 9, z = 60, Figure 1B) was determined on the basis of the group average of fMRI activity in a previous study (performed with different subjects) that showed pre-SMA is involved in accelerated speech processing (Dietrich et al., 2013a). The control stimulation site in left MOG (MNI coordinates x = −44, y = −74, z = 6, Figure 1B) was adopted from studies that similarly used MOG as a control area for speech experiments (Restle et al., 2012; Murakami et al., 2015). These authors investigated the effect of cTBS on speech-related MEPs during a passive listening task. Regarding these studies, stimulation was applied to target sites of the left dorsal and ventral auditory stream within IFG and temporal lobe. In order to investigate the topographical specificity of cTBS effects, they targeted left MOG as control site, which is involved in visual processing, but is not considered as part of the auditory speech-processing network (Restle et al., 2012; Murakami et al., 2015).

These coordinates were identified on each subject's brain by using a TMS neuro-navigation system (Localite GmbH, Sankt Augustin, Germany). The individual structural T1-weighted image (3D high-resolution) was imported and MNI target coordinates were transformed to individual ones. For spatial calibration, five skull landmarks (nasion, bilateral corners of the eyes, and bilateral pre-auricular points) and 200 points on the surface of scalp of each subject were fitted to the 3D image. Errors between the subject's scalp and the image were allowed within 3 mm. The stimulating coil was visually navigated to the stimulation site and kept there on the basis of real time feedback of the coil position throughout the cTBS application. CTBS was applied through a biphasic magnetic stimulator (Magstim Super Rapid) and a 70 mm figure-of-eight stimulating coil (Magstim Company, UK). In line with Huang et al. (2005), 600 pulses were applied in a theta burst-pattern (bursts of three pulses at 50 Hz, repeated at 5 Hz for a duration of 40 s). By adopting the approach of Mars et al. (2009), stimulation intensity was adjusted to 120% resting motor threshold (RMT) for the abductor pollicis brevis muscle of the right hand. RMT was defined as the minimum stimulator output which was required to produce motor-evoked potentials (MEP) >50 μV peak-to-peak amplitude in at least 5 out of 10 consecutive trials at the optimal scalp position overt left motor cortex (Rossini et al., 2015). According to a previous study (Grossheinrich et al., 2009) a stimulation intensity of 80% RMT of the tibialis anterior muscle for cTBS over the medial prefrontal cortex (electrode position Fz = midline frontal) can be considered safe. Given that the RMT of lower limb muscles is considerably higher than that of small hand muscles by a factor of about 1.6 (Chen et al., 1998), a stimulation intensity of 120% RMT of the abductor pollicis brevis muscle applied over the pre-SMA was considered to be safe for cTBS in the present study. The average stimulation intensity (120% of RMT) was 46.2% of maximum stimulator output (range 40%−58%) in the experimental group (pre-SMA). In the control group (MOG) a stimulation intensity of 40% of maximum stimulator output (lowest value of the experimental group) was used for all subjects. This low intensity was used because during stimulation of MOG some subjects reported strange feelings and/or pain around the stimulated region (temporal muscle) when RMT was in the range defined individually for a subject. The stronger stimulus intensity for pre-SMA as compared to MOG might confound the results to some extent. However, a more serious influence would have been expected by irritating nerve stimulation in case of high MOG stimulation intensities. The coil was placed tangentially over the left pre-SMA, with the coil handle pointing backwards at an angle of 45° to the anterior-posterior axis. Concerning the left MOG, the coil handle pointed downwards parallel to the vertical crossing of the anterior commissure. Thus, direct stimulation of the temporal muscle could be avoided.

### Evaluation of behavioral data

Based on the audio recordings, the repetition trials were orthographically transcribed. Word by word, the utterances were categorized as correct when the production was identical or nearly identical such as in case of a deviant singular/plural endings (e.g., “Flugzeug”—“Flugzeuge”), deviant tense (e.g., “soll”—“sollte”), or deviant (short) prefix (e.g., “um-geworfen”—“geworfen”). In these cases, the syllables of the target were judged as correct (see Supplementary Table 2 for examples). Incorrect responses were categorized as errors that could either be “unrelated” or “related” to the target words. Thereby, items showing semantic and/or phonological similarity to the target word were categorized as related whereas all other errors were considered as unrelated. Semantic relatedness comprises synonyms (e.g., “Fest”—“Feier”), words belonging to the target word field (e.g., “Pferde”—“Schafe”), substitution of definite and indefinite articles or possessive pronouns (e.g., “eine”—“die”—“ihre”), separated or fused prepositions (e.g., “bei dem”—“beim”), semantically plausible prefix addition or substitution (e.g., “um-geworfen”—“herunter-geworfen”), substitution of auxiliary verbs (e.g., “muss”—“soll”), and deviant gender of pronouns (e.g., “er”—“sie”). Phonological relatedness was determined by overall phonological similarity such as shared phonological features with the target, e.g., p/b, p/f, l/r, p/t, substitution of only single vowels while at least half of syllables of a response word was identical or nearly identical with the target (e.g., “Künstlerin”—“Kanzlerin”). In case that in a reproduced word syllables of a target word were missing while the remaining part showed phonological similarity (e.g., “Marathon”—“Mal”), the reproduced syllables were counted as related (for further examples see Supplementary Table 2). Missing words, which were not substituted by an incorrect word, were counted as silent events weighted with the number of syllables within these words (missing minus incorrect = silent). In case of negative values (more incorrect than missing target syllables) the silent value was set to zero. Finally, qualitative aspects of incorrect repetitions, i.e., the percentage of unrelated errors (based on the total amount of incorrect errors), indicating the monitoring functions with respect to plausibility, were analyzed.

The person evaluating individuals' responses was blinded, i.e., the time points of measurement (pre/post1/post2) were not disclosed. In order to assess inter-rater reliability, a second person additionally evaluated responses of 11 subjects from the experimental group (pre-SMA). Inter-rater reliability was acceptable as determined by Cronbach's alpha with respect to the number of syllables given to each parameter within each sentence (correct repetitions α = 0.986, silent events α = 0.984, incorrect repetitions α = 0.967, unrelated errors α = 0.933, related errors: α = 0.843).

### Statistical analysis

First, in order to describe alterations of speech comprehension as a function of speech rate, the distributions of parameters—correct and incorrect responses, silent events, unrelated, and related errors—were plotted against the five speech rates (8, 10, 12, 14, 16 syl/s). Data of each speech rate were pooled across all measurement time points (pre, post1, post2) and across the experimental (pre-SMA) and control (MOG) group. Repetition performance was quantified as the proportion of correct, incorrect (related plus unrelated), and silent events, which additively resulted in 100% (= 18 syllables per sentence). Numeric values are listed in Table 1.

**Table 1:** Number of syllables.

	**8**	**10**	**12**	**14**	**16**	**All rates**
**PRE-SMA: CORRECT REPETITIONS**						
Pre	158.00 (6.46)	157.18 (6.28)	137.29 (16.56)	112.41 (18.29)	67.11 (25.67)	632.00 (60.43)
Post1	158.11 (4.73)	156.83 (6.44)	132.61 (14.72)	108.83 (22.23)	63.00 (27.27)	619.39 (62.10)
Post2	158.61 (4.74)	154.89 (6.73)	144.17 (8.94)	113.50 (25.80)	77.72 (20.43)	648.89 (52.23)
**PRE-SMA: SILENT EVENTS**						
Pre	0.82 (1.42)	2.82 (4.00)	19.71 (15.66)	39.59 (19.19)	82.00 (27.23)	147.49 (60.35)
Post1	1.94 (3.76)	2.94 (3.81)	21.89 (12.82)	43.17 (19.68)	85.67 (30.68)	155.61 (58.73)
Post2	1.94 (2.92)	4.94 (4.61)	13.17 (6.57)	38.22 (24.40)	72.50 (22.34)	130.78 (48.55)
**PRE-SMA: INCORRECT REPETITIONS (** = **UNRELATED** + **RELATED)**						
Pre	1.29 (2.49)	2.12 (2.69)	5.18 (4.28)	10.24 (5.65)	13.12 (5.37)	31.94 (12.56)
Post1	2.50 (2.36)	2.50 (3.73)	7.72 (6.22)	10.56 (6.97)	13.44 (9.90)	36.72 (22.65)
Post2	1.61 (2.12)	2.56 (2.79)	5.06 (4.05)	10.78 (8.69)	12.06 (5.81)	32.06 (17.92)
**PRE-SMA: UNRELATED ERRORS**						
Pre	0.35 (0.79)	0.82 (1.33)	2.77 (2.51)	5.06 (4.52)	6.71 (4.06)	15.71 (7.59)
Post1	1.56 (2.04)	1.78 (2.44)	4.00 (4.67)	7.00 (5.03)	8.83 (7.09)	23.17 (16.06)
Post2	0.83 (1.58)	1.28 (1.36)	2.39 (1.94)	5.67 (5.93)	6.44 (4.45)	16.61 (12.02)
**PRE-SMA: RELATED ERRORS**						
Pre	0.94 (2.25)	1.29 (1.99)	2.41 (2.40)	5.18 (3.81)	6.41 (2.69)	16.24 (7.69)
Post1	0.94 (1.43)	0.72 (1.71)	3.72 (2.95)	3.56 (2.91)	4.61 (4.60)	13.56 (8.26)
Post2	0.78 (1.48)	1.28 (2.27)	2.67 (2.57)	5.11 (4.84)	5.50 (2.64)	15.33 (7.96)
**MOG: CORRECT REPETITIONS**						
Pre	158.89 (3.10)	155.94 (4.94)	132.39 (15.66)	108.22 (20.02)	56.06 (23.63)	611.50 (50.17)
Post1	158.06 (6.79)	155.78 (8.83)	139.22 (12.76)	110.06 (23.42)	62.39 (20.14)	625.50 (52.98)
Post2	160.50 (1.79)	158.00 (4.01)	130.39 (18.31)	117.28 (20.01)	71.33 (19.66)	637.5 (49.72)
**MOG: SILENT EVENTS**						
Pre	1.78 (2.02)	4.89 (4.55)	23.61 (13.89)	44.50 (18.78)	94.56 (22.97)	169.33 (48.30)
Post1	1.94 (3.15)	3.94 (6.58)	17.28 (12.00)	43.89 (22.70)	88.94 (22.02)	156.00 (51.32)
Post2	0.61 (1.24)	2.61 (3.66)	24.72 (14.89)	37.11 (17.62)	79.17 (17.75)	144.22 (43.42)
**MOG: INCORRECT REPETITIONS (** = **UNRELATED** + **RELATED)**						
Pre	1.61 (2.30)	1.61 (2.45)	6.44 (5.16)	9.67 (6.71)	11.50 (7.83)	30.83 (14.45)
Post1	1.22 (2.02)	2.61 (3.60)	5.78 (4.24)	8.67 (6.42)	10.89 (7.98)	29.17 (17.39)
Post2	0.89 (1.13)	2.11 (2.99)	7.39 (7.45)	8.00 (6.11)	11.67 (8.05)	30.06 (19.06)
**MOG: UNRELATED ERRORS**						
Pre	0.56 (1.29)	0.83 (1.38)	3.61 (4.05)	5.89 (4.36)	7.33 (6.59)	18.22 (11.08)
Post1	0.50 (0.86)	1.06 (2.29)	2.67 (2.61)	4.94 (4.39)	5.78 (5.99)	14.94 (12.02)
Post2	0.67 (1.03)	1.33 (2.61)	4.17 (5.17)	4.11 (3.88)	7.44 (6.81)	17.72 (13.35)
**MOG: RELATED ERRORS**						
pre	1.06 (1.70)	0.78 (1.48)	2.83 (2.55)	3.78 (3.26)	4.17 (2.79)	12.61 (6.45)
Post1	0.72 (1.64)	1.56 (2.09)	3.11 (3.07)	3.72 (4.18)	5.11 (3.32)	14.22 (7.20)
Post2	0.22 (0.55)	0.78 (1.17)	3.22 (3.37)	3.89 (3.51)	4.28 (2.80)	12.39 (7.25)

*Syllables resulted from nine sentences per speech rate (= 45 sentences) were summed and afterwards averaged across subjects (n = 18). Mean and standard deviation (in parenthesis) for the experimental (pre-SMA) and control (MOG) stimulation site. Slight inaccuracies of the sum (162 syllables for each rate or 810 syllables across all rates) of correct, silent, and incorrect events resulted from incorrect syllables more than substituted for the target*.

**Figure 2:**
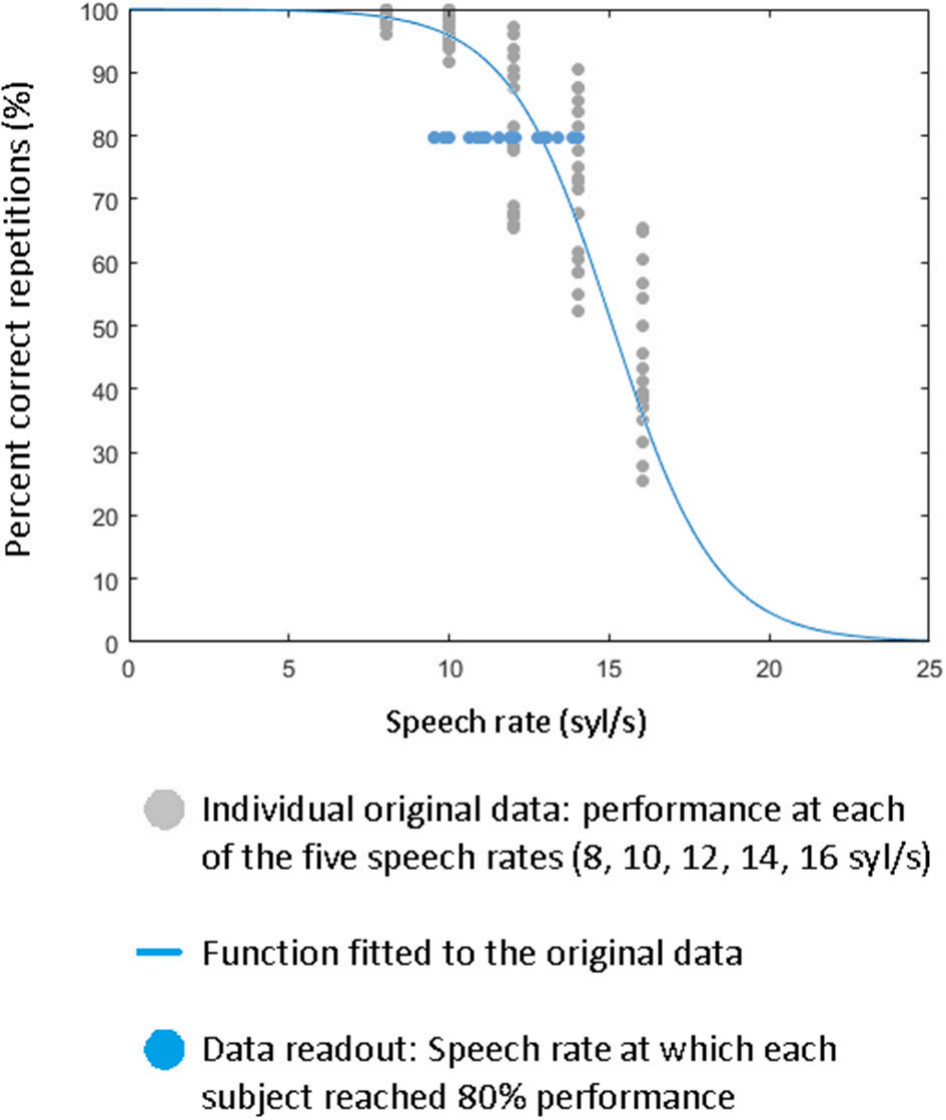
Percentage of correctly reproduced speech material as a function of syllable rate (8, 10, 12, 14, 16 syl/s), fitted to a psychometric function (exemplified for a single subject = blue curve), Blue dots correspond to the individual syllable rate at which subjects' performance of speech comprehension amounts 80%, as determined by the psychometric function. Each data point corresponds to a single subject (exemplified for the pre-baseline condition of the experimental subject group).

In order to obtain a single estimate of the overall performance (correct repetitions) across all speech rates (quantitative analysis), a psychometric function (Wichmann and Hill, 2001a,b) was fitted to the percentage of correct syllables across the five speech rates (Figure 2), and from this function the syllable rate with correct reproduction of 80% was determined (Figure 2). The 80% value was chosen because at this point speech comprehension is still present, but under time-critical circumstances, requiring the hypothesized function of pre-SMA.

Statistical assessment of the cTBS effect was performed by means of a repeated measure ANOVA using the within-subject factor time (pre/post1/post2) and the between-subject factor site (SMA/MOG). This kind of analysis was applied to the overall performance (= syllable rates at 80% correctly reproduced words). Thereby, statistical analysis was based on three a priori assumptions: (i) Since a pre/post1/post2 design was used predicting the effect of cTBS after post1 compared to baseline measurements (pre/post2), a quadratic relationship was strongly expected. (ii) Further, since an inhibitory TMS protocol was applied, reduction of performance (“dip” of the quadratic function) was predicted justifying one-tailed testing of the quadratic relationship of time. (iii) Further, transient reduction of speech performance was expected exclusively for the pre-SMA stimulation site, while stimulation of MOG, which was selected as speech irrelevant control area (Restle et al., 2012; Murakami et al., 2015), should not lead to a reduction in speech performance. Therefore, statistical analyses focused on the interaction time × site, expected to be significant with a quadratic relationship of time. Since the approach provided clear a priori justifications given by the design (post1), protocol (“dip”), and control area (MOG), we skipped the first analysis of global effects (tests out of interest, many degrees of freedom concealing possibly effects of interest) and directly considered the interaction of interest. Additionally, comparing directly pre- to post1-measurements after pre-SMA and MOG stimulation, a repeated measures ANOVA with the inner-subject factor time (pre/post1) and the between subject factor site (pre-SMA/MOG) was conducted. Thereby, the difference between pre- and post2-measurement was added as covariate controlling for baseline variables.

Furthermore, investigating the hypothesis that TMS-induced suppression of overall performance depends on speech rate, a repeated measures ANOVA with the factors time, site, and rate (with the levels low rates = 8, 10 syl/s and high rates = 12, 14 syl/s) was applied. Thereby, percent values of syllables within correctly reproduced words (based on maximal 18 syllables per sentence) were averaged across low (8, 10 syl/s) and high (12, 14 syl/s) speech rates. We expected a significant three-way interaction between time × site × rate indicating a stronger TMS-induced reduction of performance at high as compared to low speech rates.

In order to obtain a second, qualitative parameter of performance a differential analysis of erroneous speech material was performed. To these ends, the percentage of unrelated errors based on the total number of incorrect syllables were analyzed (the sum of unrelated and related errors resulted in the number of incorrect repetitions). This analysis was performed on pooled data across all five speech rates, since incorrect repetitions showed only few events within single speech rates. As concerns the qualitative analysis (percentage of unrelated errors), we expected an increase of unrelated errors after cTBS (post1) compared to the baseline measurements (pre, post2) after pre-SMA stimulation. Additionally, comparing directly pre- to post1-measurements after pre-SMA and MOG stimulation, a repeated measures ANOVA with the inner-subject factor time (pre/post1) and the between subject factor site (pre-SMA/MOG) was conducted. Thereby, the difference between pre- and post2-measurement was added as covariate controlling for baseline variables. Effect sizes for the pre-post1 comparisons were given as Cohen's d.

Values of each parameter (syllable rates at 80% correct repetitions; percent correct repetitions [based on max. 18 syllables per sentence] at low (8, 10 syl/s) and high rates (12, 14 syl/s); percentage of unrelated errors [based on the total number of incorrect repetitions]) were tested for normal distribution (Shapiro Wilk's test). If normal distribution could not be assumed, non-parametric testing was used. Furthermore, the repeated measures ANOVA was validated with respect to the within subject factor time (Mauchly's sphericity) and the between-subject factor site (Levene's test).

## Results

### Descriptive analyses and baseline effects

**Figure 3:**
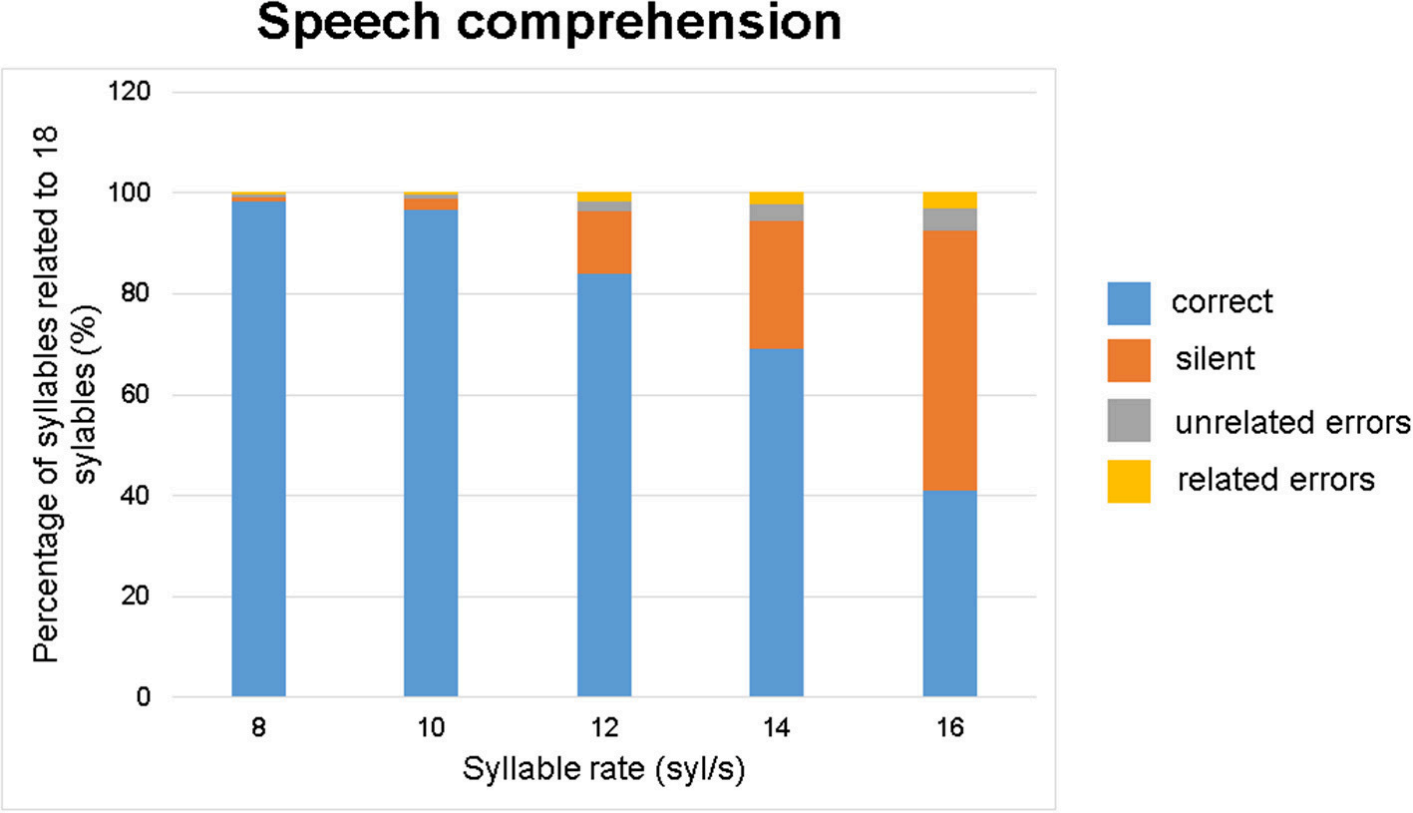
Speech comprehension as a function of speech rate: percentage of correct, silent, or erroneous (unrelated and related) speech material. Data were pooled across all measurement time points (pre, post1, post2) and subjects.

As shown in Figure 3, the overall performance (percentage of correctly reproduced material) strongly decreased while incorrectly reconstructed words or silent events increased with speech rate. On average (across all conditions, speech rates, and stimulation sites), correct repetitions amounted to ca. 78%, silent events to ca. 18% and incorrect (related and unrelated) reproductions to ca. 4%. Absolute numbers of syllables are listed in Table 1 for each of the three measurement time points (pre/post1/post2) and for the experimental and control stimulation site.

Normal distribution was asserted using Shapiro Wilk's Test (*p* > 0.05) which showed that normality could be assumed in each stimulation site (pre-SMA/MOG) and each time point of measurement (pre/post1/post2) with respect to the syllable rate at 80% correct repetitions, percentage of correct repetitions at high speech rate, and percentage of unrelated errors. The percentage of correct repetitions at low speech rates did not reach normal distribution, caused by the ceiling effect (almost 100% performance in each subject). Variances between stimulation sites were found to be homogeneous (*p* > 0.05) and sphericity of the factor time was given in all parameters (*p* > 0.05).

### cTBS effects on correct repetitions

**Figure 4:**
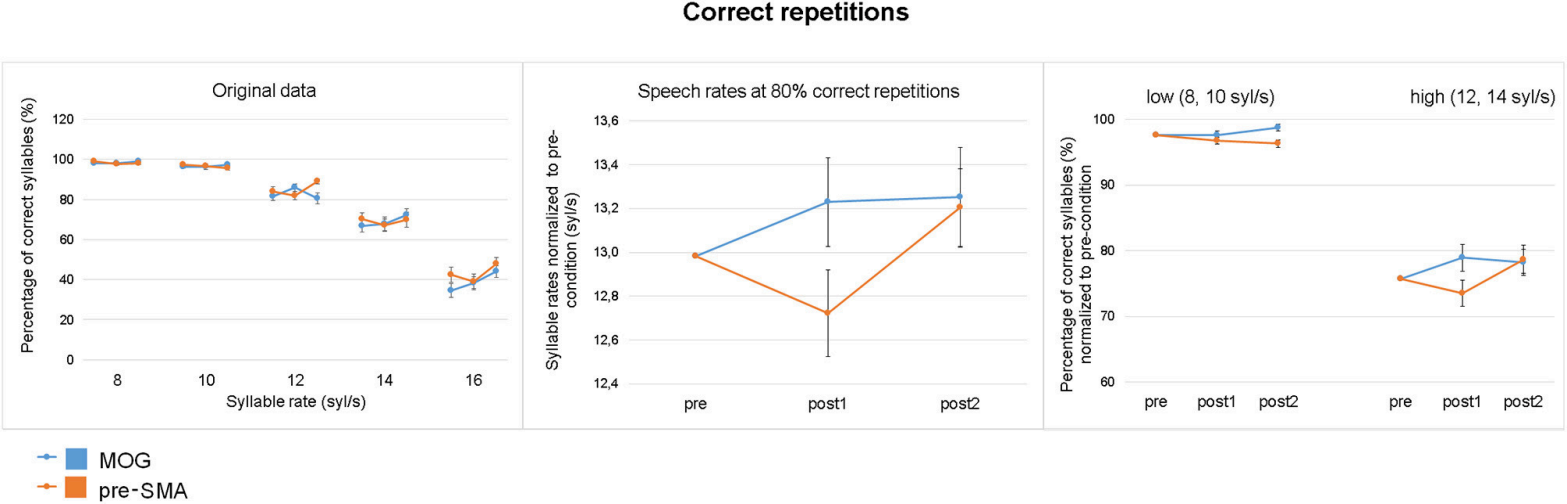
Effect of cTBS on correct repetitions. **(Left)** Percentage of correctly reproduced syllables (based on 18 syllables per sentence) across the five speech rates for the experimental (pre-SMA) and control (MOG) stimulation site during pre-, post1-, and post2-measurements (mean, standard error). **(Middle)** Performance in terms of the syllable rate at which 80% of the speech material was correctly reproduced (as determined with the psychometric function), normalized to the grand average of the pre-condition (mean, standard error). **(Right)** Percentage of correct repetitions (mean, standard error, normalized to the grand average of the pre-condition) averaged across slow (8, 10 syl/s) and fast (12, 14 syl/s) speech rates. Stimulation over pre-SMA caused a transient reduction of overall performance (middle) which was due to reduced comprehension of fast speech (right).

As expected, speech comprehension declined at high syllable rates (Figure 4 left). Descriptively, stimulation of pre-SMA caused a transient reduction of performance at syllable rates of 12 syl/s or faster, indicated as a “dip” in the post1 as compared to pre and post2 runs, which was absent in the control group (Figure 4 left). Performance of speech comprehension (= syllable rate at which 80% of the stimulus text could be reproduced) revealed a significant interaction time × site with respect to the quadratic relationship of time [*F*_(1, 34)_ = 3.614, *p* = 0.033, one-tailed]. As hypothesized, performance was significantly reduced after cTBS over pre-SMA (“dip”) [*F*_(1, 17)_ = 5.472, *p* = 0.032], whereas cTBS over MOG did not show such an effect [*F*_(1, 17)_ = 0.251, *p* = 0.623; Figure 4 middle]. Quantifying the “dip” in speech comprehension, 72.2% of individuals participating the experimental group (pre-SMA) showed negative values (subtracting the mean of pre and post2 from post1) indicating reduction of speech comprehension whereas in the control group (MOG) the percentage of individuals showing negative values was only 38.8%. Regarding the experimental group (pre-SMA), mean syllable rate at which subjects showed 80% correct sentence repetition was 13.16 syl/s (*SE* = 0.25) for the pre-baseline, 12.87 (0.24) for the post1 test measurement, and 13.37 (0.26) for the post2-baseline. The respective values for the control group (MOG) were pre: 12.80 (0.21), post1: 13.03 (0.24), and post2: 13.06 (0.28). Although visual inspection of the results (Figure 4 middle) suggests a group difference, the main effect of site was not found to be significant [*F*_(1, 34)_ = 0.318, *p* = 0.577]. Furthermore, the interaction time × site with a linear trend of time (slight increase in performance from pre to post2) was not significant [*F*_(1, 34)_ = 0.025, *p* = 0.875]. Direct comparisons between pre- and post1 measurement (pre-, post2 differences as covariance adjustment) revealed a significant interaction time × site [*F*_(1, 33)_ = 3.605, *p* = 0.033, one-tailed] as well as a significant interaction time × covariate [*F*_(1, 33)_ = 6.666, *p* = 0.014] indicating that the covariate ran counter to the pre-post1 effect. *Post hoc* the factor time as well as the interaction time × covariate revealed significant effects after pre-SMA [time: *F*_(1, 16)_ = 5.657, *p* = 0.030, *d* = 0.3; time × covariate: *F*_(1, 16)_ = 7.486, *p* = 0.015], but not after MOG stimulation [time: *F*_(1, 16)_ = 0.624, *p* = 0.441, *d* = 0.2; time × covariate: *F*_(1, 16)_ = 1.501, *p* = 0.238]. A main effect of covariate was not found to be significant.

Regarding the differential TMS effect on high vs. slow speech rates, the three-way interaction time × site × rate was found to be significant with a quadratic trend of time [*F*_(1, 34)_ = 5.213, *p* = 0.029] indicating a reduction of performance after cTBS at high [*F*_(1, 34)_ = 5.386, *p* = 0.026), but not at low speech rates [*F*_(1, 34)_ = 0.280, *p* = 0.600]. Further, the factor time with a quadratic trend was found to be significant at high speech rates after pre-SMA stimulation [*F*_(1, 17)_ = 6.726, *p* = 0.019], but not after MOG stimulation [*F*_(1, 17)_ = 0.226, *p* = 0.640, Figure 4 right].

Since, data of slow rate conditions were not normally distributed caused by a ceiling effect (subjects understood almost 100%), effects were additionally analyzed by a non-parametric test. Thereby, considering low speech rates the three time points (pre/post1/post2) did not differ after pre-SMA stimulation (Friedman test, X^2^ = 2.735, *p* = 0.255), while a significant effect could be observed in the control (MOG) condition (Friedman test, X^2^ = 6.206, *p* = 0.045). The latter was related to pre-post2 (baseline) differences (Wilcoxon test post2 vs. pre: X^2^ = −2.155, *p* = 0.031 not reaching Bonferroni correction). However, regarding high speech rates, significant differences between the three time points were found after pre-SMA (Friedman test, X^2^ = 7.111, *p* = 0.012, one-tailed), but not after MOG stimulation (X^2^ = 0.592, *p* = 0.744). *Post hoc*, after pre-SMA stimulation significant differences could be found between pre and post1 as well as post1 and post2 conditions (Wilcoxon test, pre vs. post1: X^2^ = −1.677, *p* = 0.047, *d* = 0.3, one-tailed, post2 vs. post1: X^2^ = −2.636, *p* = 0.008, *d* = 0.5). Direct comparisons between stimulation sites (pre-SMA vs. MOG) with respect to pre and post1 differences were found to be larger under the experimental than control condition at high speech rates (Mann-Whitney U test: U = 91, *p* = 0.025, *d* = 0.7).

### cTBS effects on a specific error type—unrelated errors

**Figure 5:**
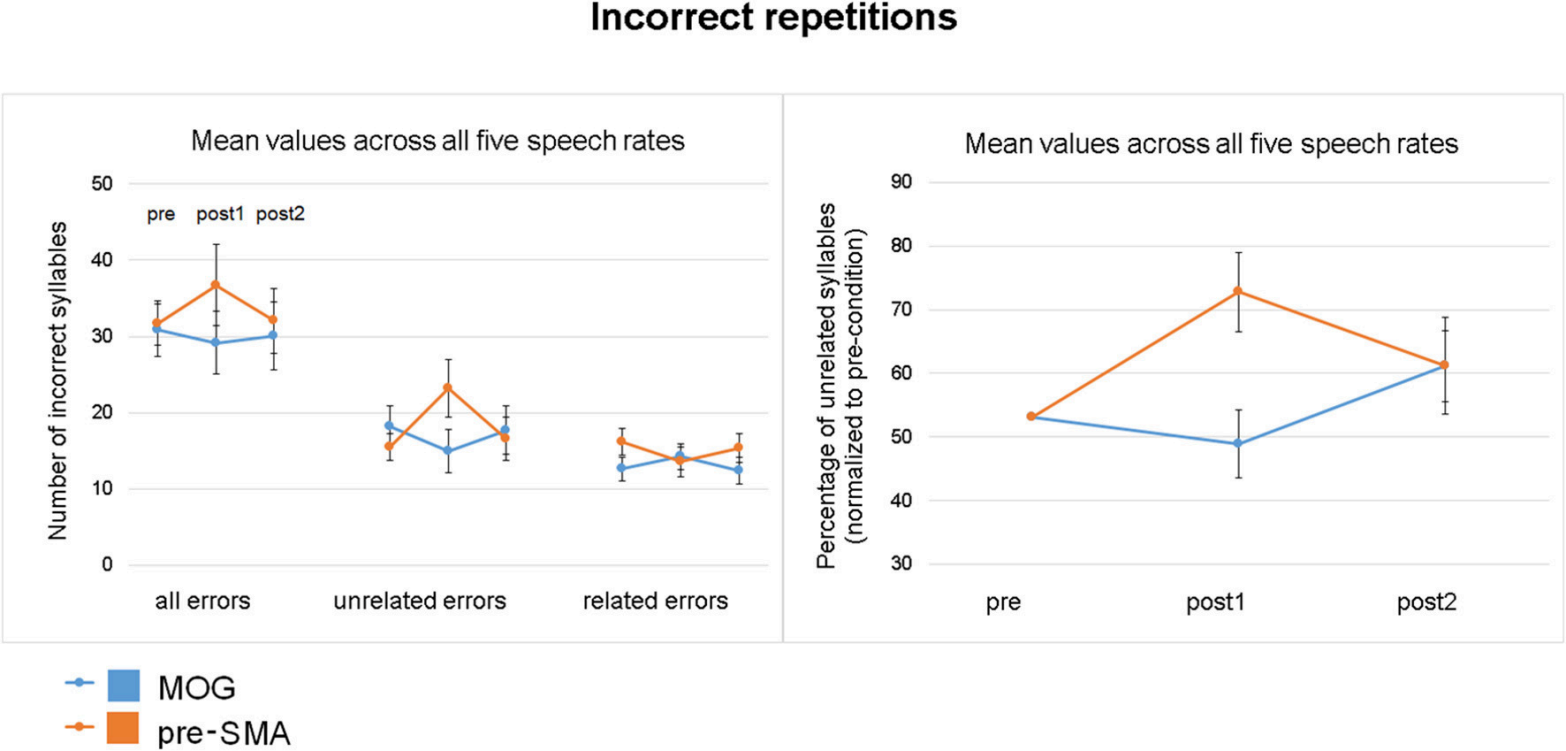
Effect of cTBS on unrelated errors (pooled across all five speech rates). **(Left)** Number of all incorrect repetitions, unrelated, and related errors plotted for the three time points of measurement (pre/post1/post2) and the two stimulation sites (pre-SMA/MOG). **(Right)** CTBS effect on the percentage of unrelated errors (based on all incorrect repetitions) for each stimulation site (pre-SMA/MOG) normalized to the grand average to pre-measurements (mean, standard error). Transient inhibition of pre-SMA resulted in an increase of unrelated errors.

As concerns the percentage of unrelated errors (based on the total amount of incorrect repetitions), a significant two-way interaction time × site with a quadratic relationship of time [*F*_(1, 34)_ = 12.830, *p* = 0.001] indicated a transient increase of unrelated errors after pre-SMA stimulation [*F*_(1, 17)_ = 5.696, *p* = 0.029], but not after cTBS over MOG (Figure 5). Quantifying the cTBS effect on unrelated errors, 72.2% of individuals of the experimental and 28.7% of subjects of the control group showed positive values (subtracting the mean of pre and post2 from post1) indicating transient increase of unrelated errors. Direct comparisons between pre- and post1 measurement (pre-, post2 differences as covariance adjustment) revealed a significant interaction time × site [*F*_(1, 33)_ = 12.422, *p* = 0.001] as well as a significant interaction time × covariate [*F*_(1, 33)_ = 19.131, *p* = 0.000]. A main effect of covariate was not found to be significant. *Post hoc* unrelated errors significantly increased during post1 measurement compared to pre-condition after pre-SMA stimulation (T = −2.470, *p* = 0.024, *d* = 0.9), while MOG stimulation did not show any significant effects (T = 1.855, *p* = 0.081, *d* = 0.6).

## Discussion

As hypothesized, a transient “virtual lesion” in the pre-SMA resulted in reduced sentence repetition performance. Transient reduction of performance was found to be significant at 80% correct repetitions, i.e., reduction of syllable rates from 13.2/13.4 (pre/post2) to 12.9 syl/s (post1). Thereby, the 80% threshold guarantees speech perception under high demand, while still enough is understood. Further, the cTBS effect was found for fast (12, 14 syl/s), but not for moderate syllable rates (8, 10 syl/s). As concerns qualitative aspects, unrelated errors significantly increased after cTBS over pre-SMA. The results did not show any change in performance from pre- to post2-baselines.

### Task difficulty—reconstruction and prediction

We used cTBS to induce a transient disruption of cortical processing in pre-SMA to gain knowledge about the functional role of pre-SMA in speech comprehension under time-critical circumstances. Hearing accelerated formant-synthesized speech of single sentences is a quite artificial condition. However, the mechanisms of phonological/lexical encoding have been suggested to be still similar to normal speech, and task difficulty (in terms of speech rate) was easy to manipulate under these conditions. Although natural speech represents “real life speech comprehension” and synthesized speech sounds a little unfamiliar, formant synthesis, due to its simple rule-based structure seems to have even some advantages regarding intelligibility at high syllable rates. This has been shown for blind subjects who use accelerated speech for text reception by comparing formant synthesis to accelerated natural speech (Moos and Trouvain, 2007) or to natural sounding diphone synthesis (Trouvain, 2007).

In the present data, transient impairment of speech comprehension was only observed for high speech rates of 12 syl/s or faster. In line with these findings, pre-SMA was found to be stronger activated in fMRI studies during presentation of ultra-fast as compared to moderately fast speech (Dietrich et al., 2013a,b) and to be particularly active near the limit of intelligibility (Vagharchakian et al., 2012). An effect of task difficulty on pre-SMA activation was also found in case of degraded speech in a sentence matching task (Clos et al., 2014a,b) or in experiments on switching between native and foreign speech perception (de Bruin et al., 2014). Thus, pre-SMA seems to be generally involved in speech processing in case of high task demands.

The present pre-SMA location for stimulation was taken from a previous study (Dietrich et al., 2013a), which was conducted on five late-blind participants and one sighted subject trained to comprehend ultra-fast speech at 16–18 syl/s. Thereby, all subjects showed extended frontal and premotor activation, i.e., pre-SMA and left IFG, after training (Dietrich et al., 2013a). Blind subjects were found to use additional strategies for accelerated speech comprehension at the sensory level, which could not be used by sighted subjects (recruitment of primary visual cortex in order to detect speech features). However, dealing with the “frontal bottleneck” of speech perception, blind and sighted subjects seem to be similar. This bottleneck, including functions of the pre-SMA, comprises the coordination of memory representations with the temporal event structure (Kotz and Schwartze, 2010) and seems to be involved in the buffering of phonological materials (Dietrich et al., 2013a).

Although the functional role of pre-SMA seems to be evident from the present data as well as previous fMRI data, its differential contribution to the entire process of speech processing should be considered more in detail. Presumably, time-critical speech perception cannot totally be performed in a bottom-up mode. Pre-SMA involvement during perception of sentence stimulus materials and adverse listening conditions could be explained by the assumption that procedural representations may contribute to disambiguate linguistic information when lexical/semantic access is difficult. Thereby, it is hypothesized that these procedural representations are linked to predictive top-down mechanisms (Hertrich et al., 2016). Utilizing general redundancy in speech and language, the speech generation mechanism can make predictions for upcoming speech material in order to save time during lexical access. Similarly, when part of the speech signal is unintelligible, a reconstruction of missing information has to be performed. In both cases, the top-down generated data stream has to be synchronized with the bottom-up information stream of the incoming speech signal. This temporal adjustment can be performed on the basis of the prosodic structure of speech, e.g., syllable rhythm, which is predominantly represented in the right hemisphere, but also on the basis of phonological and semantic content kept in the verbal working memory represented in the left hemisphere (Ross, 1981; Gorelick and Ross, 1987; Ross and Monnot, 2008; Friederici and Gierhan, 2013).

Various studies document pre-SMA involvement in repair mechanisms occurring under high demand conditions (Scott et al., 2004; Lima et al., 2016 for a review; Adank and Devlin, 2010). Similarly, the current results showed an effect of pre-SMA stimulation only when the task requires more effort/attention (high speech rates). Actually, reconstruction of missing materials (in the auditory representation) becomes only necessary under high demand conditions. Thus, the increased effort (requiring pre-SMA) seems to reflect the necessity of using predictive top-down mechanisms. Regarding the timing of motor events, the right hemisphere has an inhibitory control function on left-dominant forward action control, working as a kind of “brake” (Aron et al., 2014). Similar control mechanisms may be present for predictive inner speech generation in the absence of any overt motor activity. Evidence for the involvement of pre-SMA in predictive language mechanisms has been provided in review papers emphasizing the role of pre-SMA as an output region from cerebellar-thalamic and basal ganglia-thalamic circuits enhancing temporal processing such as interval estimation and extraction of temporal regularity (Schwartze et al., 2012a,b). Once temporal regularity is perceived, context-based predictions would allow the system to reconstruct omitted events (Kotz and Schwartze, 2010). Previous studies showed that syllable onsets are tracked at the sensory level (Hertrich et al., 2013), presumably, in order to predict the timing of the next incoming item. To facilitate lexical access of the incoming signal, the anticipatory timing of syllable onsets has to be imposed on predicted phonological chunks. Thus, conceivably, in addition to timing information, content-related predictions based on articulatory-phonological as well as lexico-semantic information might help to overcome the difficulty of accelerated speech comprehension. Since the function of pre-SMA was disturbed, consistent predictions/reconstructions (= successful adjustment of timing and content) could no longer be signaled to the speech generation system (left IFG) in order to reproduce the sentence correctly.

Monitoring or anticipation of articulatory gestures, i.e., access to motor representations without execution, i.e., inner speech, seems to be an effective way for speech perception under high demand such as high speaking rates (Hertrich et al., 2016). In the actual study, perception could not be separated from production since perception was tested by a repetition task. However, comparing high and slow speech rates, the cTBS effect occurred on high, but not on low speech rates. If pre-SMA inhibition had an impact on speech production instead of perception, the TMS effect would be observable under high as well as slow speaking rates. Since this was not the case, inhibition of pre-SMA was suggested to be relevant to speech perception. However, the repetition task force participants to perform a sensory-to-motor transformation in the way that syllable perception might also include the storage of the articulatory gestures. Thus, during perception of low syllable rates articulatory gestures are clearly represented requiring no further inhibitory process from pre-SMA during production. If the motor plans are not represented clearly during perception (high rates), pre-SMA needs to more strongly inhibit wrong motor plans during production. Based on the current results, this possibility cannot be excluded. In other words, when sentences are more difficult to understand, the planning of speech production will also be more challenging, e.g., participants could be unsure about which words to be produce. Thus, it is obvious that the repetition task chosen in the current study makes it difficult to separate aspects of perception from production with respect to the “planning” function. As concerns direct motor execution (not planning) of repetition, SMA proper would be an appropriate candidate for controlling this function (Picard and Strick, 1996). However, the comprehension of sentences generally requires inner speech mechanisms including monitoring and phonological planning stages, irrespective of whether a motor response (repetition task) is required or not. Lima et al. (2016) discussed strong functional (and structural) connections between pre-SMA and SMA proper enabling auditory perception and auditory imagery. Inner speech could be considered as an imagery of speech sounds initiated by the mental representation of articulatory gestures. Thus, the present findings are in line with Lima et al.'s hypothesis 2016 that pre-SMA is involved in planning and/or monitoring inner speech. It cannot be completely excluded that the present pre-SMA stimulation also affected the motor action of the repetition task (rather than reconstruction and monitoring). Maybe this could be shown by an analysis of speech motor characteristics such as articulator velocities, but this was not measured in the present study. Nevertheless, since previous studies reported strong activation of pre-SMA during passive listening to accelerated speech (Vagharchakian et al., 2012; Dietrich et al., 2013a,b), concomitant with activation of left IFG, the present effect of pre-SMA stimulation may indicate an involvement of the speech generation system in the process of speech perception rather than an impairment of motor output.

### Inhibitory control mechanism

Pre-SMA activity was observed in monkeys when they had to discard a current motor plan and acquire a new plan for future performance (Shima et al., 1996; Tanji, 1996). In humans, a frontal inhibitory control (“no go”) network has been outlined comprising, especially, pre-SMA and right-hemispheric inferior frontal cortex (Sharp et al., 2010; Swann et al., 2012; Aron et al., 2014). In case of erroneous response selection (i.e., detection of an implausible item that does not fit into the context), the ongoing top-down modulated information stream must be interrupted and restarted in order to avoid further misunderstandings when the system is “on the wrong track”. In the absence of such an inhibitory mechanism, i.e., after suppression of pre-SMA, implausible alternatives will no longer be inhibited as shown in the present results. Evidence for the inhibitory control function of pre-SMA was also provided by intra-individual comparisons of reaction time in a stop signal task (Chao et al., 2009). In line with these findings, anodal transcranial direct current stimulation of pre-SMA resulted in enhanced inhibitory control in a stop movement task (Kwon and Kwon, 2013).

In order to integrate the proposed inhibitory mechanism during speech perception in a broader functional role of pre-SMA, tasks requiring monitoring (active construction of perceived information) should be in the focus, particularly with respect to the inhibition of implausible alternatives that are not in the range of expectations. Anticipation of sequences (e.g., music) or scenarios (e.g., face-to-face communication) might enable effective/complete and fast/automated comprehension of the whole over a longer time window using internal statistics. Therefore, pre-SMA may be considered as a supra-modal region translating the results of the internal statistics (verification/falsification) into potential action patterns (inhibition/passing).

### Pre-SMA and the network for speech perception

Previous studies reported a division of SMA into higher cognitive (pre-SMA) and motor-related (SMA-proper) functions (Chee et al., 1999; Moore-Parks et al., 2010). As concerns the perisylvian language network, various studies indicate a modular structure with respect to sub-functions such as articulatory-phonological vs. semantic processing (Anwander et al., 2007; Friederici, 2009; Murakami et al., 2015). The posterior-anterior organization of SMA into motor-related functions (SMA-proper) and higher-order cognitive processing (pre-SMA) is organized largely parallel, also in structural connectivity patterns, to the ventral and dorsal premotor regions (Anwander et al., 2007), with their articulatory-phonological (dorsal stream) and semantic (ventral stream) sub-functions, respectively (Hickok and Poeppel, 2007; Saur et al., 2010). Speculatively, pre-SMA might feed into these premotor or inferior frontal nodes (BA44, BA45) of the dual pathways in a selective way: anterior parts of pre-SMA regulate lexico-semantic reconstruction while posterior parts of pre-SMA link auditory speech information with related motor programs (articulatory-phonological) in order to optimize speech processing (Lima et al., 2016). Erroneous speech material of the present study was classified with respect to phonological or semantic similarity to the target. In case of semantic relatedness, reproduced words were synonyms or words belonging to the same semantic field whereas phonological similarity was characterized by a similar surface structure, irrespective of the meaning of these words. However, statistical inferences on semantic vs. phonological errors were not given any further consideration due to the small number of syllables. Furthermore, the stimulation site of the present study was not chosen to selectively influence phonological vs. semantic processing. For future studies, it might be hypothesized that stimulation of a more anterior (MNI coordinate y > 9) vs. a more posterior region (MNI coordinate y < 9)—compared to the present stimulation site (MNI coordinate y = 9)—will allow for observing differential error patterns regarding semantic vs. phonological top-down strategies, respectively: For example, stimulation of a more anterior region might result in reduction of semantically plausible alternatives while phonological plausible items remain unaffected.

In order to gain a more complete insight into sub-functions of the speech network, other stimulation sites should be considered, such as frontotemporal parts of the language network comprising the various nodes of the dorsal (phonological) and ventral (semantic) pathways. Each of these regions might lead to a specific error pattern after TMS stimulation. Nevertheless, the paradigm used in the present study has shown that manipulations of cognitive processes during speech perception are possible and that the analysis of incorrect repetition behavior provides some insight into the function of stimulated region.

### MOG—A speech-irrelevant control area?

The MNI coordinates of the control area (MOG) used in the present study correspond to occipital lobe area V5, a region which was found to be sensitive to visual motion processing (Zeki, 2015). This region is laterally surrounded (closer to the stimulation coil) by the occipital lateral area V4. Functionally, V4 is part of the ventral visual “what” pathway [running from V1 to the temporal lobe (Desimone, 1991; Tanaka, 1997)], involved in visual object identification and recognition (Zeki, 1983; Desimone and Schein, 1987). Inhibitory stimulation of V5 (MOG) and V4 might, on the one hand, reduce “visual noise” (which is normally regulated/decreased by higher cognitive mechanisms) so that the auditory system (and also speech processing) is facilitated. On the other hand, cTBS over V5/V4 might reduce speech comprehension due to the interruption of audio-visual interactions resonating within the mental lexicon (“ventral pathway” V4) and the visual imagery of articulation (V5). Regarding the overall performance neither facilitation nor any impairment could be observed after MOG stimulation compared to the baseline conditions (pre, post2).

A limitation of the current study might be the fact that, in order to avoid uncomfortable side effects of nerve stimulation, TMS intensity was lower in the control area (MOG) than in the test region (pre-SMA). Thus, it cannot be excluded that higher MOG stimulation might have an effect on speech comprehension. However, in line with previous reports in the literature (Restle et al., 2012; Murakami et al., 2015), the present results did not even show a tendency of decreased performance after MOG stimulation.

## Conclusion

Taken together, cTBS of pre-SMA reduced the performance of speech comprehension, indicating an engagement of pre-SMA in language functions. However, significant effects of cTBS of pre-SMA occurred only under time-critical circumstances, which might be explained by the assumption that in case of increased task demands additional pre-SMA-dependent top-down mechanisms are engaged, enabling prediction, and reconstruction of partially unintelligible speech materials. Thereby, pre-SMA might contribute to an integration of right-hemispheric (syllable timing) and left-hemispheric (phonological sequencing, semantic mapping) functions, eventually mediated by subcortical structures. As concerns the kind of errors being made after cTBS-induced pre-SMA suppression, implausible errors increased, suggesting that under suppression of pre-SMA implausible errors will no longer be inhibited.

## Author contributions

HA, IH, SD, UZ, and FM-D delineated the rationale and developed the design of the study. IH, SD, PB, DD, FM-D, and VS were engaged in data collection and development of analyses methods. SD and VS performed the behavioral and MRI and TMS data analyses, and drafted the first version of the paper. All authors contributed to the final version of the manuscript and approved its content.

### Conflict of interest statement

The authors declare that the research was conducted in the absence of any commercial or financial relationships that could be construed as a potential conflict of interest.
