# Supplementary material for: Reduced Performance During a Sentence Repetition Task by Continuous Theta-Burst Magnetic Stimulation of the Pre-supplementary Motor Area
Source: Front Neurosci. 2018 May 29;12:361. doi: 10.3389/fnins.2018.00361 (PMC5987029; doi:10.3389/fnins.2018.00361)
Supplement: Supplementary file 2 [file Table_2.DOCX]

**Supplementary Table 2.** Examples for evaluation of errors. The sum of correct, incorrect, and silent events resulted in 18 syllables that is 100% (see example 1). Rounding errors (see example 2) and slight inaccuracies (see example 3) resulting from numerous incorrect syllables (more than substituted for the target) exceeding the 100% value. Slightly changed words such as in example 4 were evaluated as correct considering the number of syllables of the target. The sum of unrelated, semantically, and phonologically related syllables resulted in the number of incorrect syllables (= approximately 100% caused by rounding errors).

| **Target sentence** | **Individual’s response** | **Correct**  **(% of 18 syllables)** | **Missing**  **(% of 18 syllables)** | **Incorrect**  **= related + unrelated (% of 18 syllables)** | **Unrelated**  **(% of incorrect)** | **Related**  **(% of incorrect)**  **= semantic + phonological** | **Semantically related**  **(% of incorrect)** | **Phonologically related (% of incorrect)** | **Silent**  **(% of 18 syllables)**  **= missing – incorrect (negative values will be valued as 0)** |
| --- | --- | --- | --- | --- | --- | --- | --- | --- | --- |
| 1. Die Katze hat beim Toben die Vase von der Kommode geworfen | Die Katze hat beim Toben die Vase --- --- ------- -------- runtergeworfen | 10 syl = 56%  (Die Katze hat beim Toben die Vase) | 8 syl = 44%  (von der Kommode) | 2 syl = 11% (runter-) | 0 syl = 0% | 2 syl = 100% (runter) | 2 syl = 100%  (runter) | 0 syl = 0% | 6 syl (8-2) = 33% |
|  |  |  |  |  |  |  |  |  |  |
| 2. Die Künstlerin entwirft ausgefallene Kleiderkreationen | Die ---------- Kanzlerin -------- ------------ ---------------- | 1 syl = 6%  (Die) | 17 syl = 94% (Künstlerin entwirft ausgefallene Kleiderkreationen) | 3 syl = 17%  (Kanzlerin) | 0 syl = 0% | 3 syl = 100% (Kanzlerin) | 0 syl = 0% | 3 syl = 100% (Kanzlerin) | 14 syl (17-3) = 78% |
|  |  |  |  |  |  |  |  |  |  |
| 3. Die Haustüre muss verschlossen sein, damit der Hund nicht ausbüxen kann | Die Haustüre muss verschlossen sein, damit --- ---- nicht -------- ausgewechselt werden kann | 14 syl = 78%  Die Haustüre muss verschlossen sein, damit nicht aus----kann | 4 syl = 22%  (der Hund -büxen) | 5 syl = 28%  (gewechselt werden) | 5 syl = 100%  (gewechselt werden) | 0 syl = 0% | 0 syl = 0% | 0 syl = 0% | 0 syl (4-5) = -1 syl = 0% |
|  |  |  |  |  |  |  |  |  |  |
| 4. Das Wartezimmer beim Arzt war wieder voll wartender Patienten | Das Wartezimmer ---- ---- war wieder ---- voller --------- Patienten | 13 syl = 72%  (Das Wartezimmer war wieder voll Patienten) | 5 syl = 28%  (beim Arzt wartender) | 0 syl = 0% | 0 syl = 0% | 0 syl = 0% | 0 syl = 0% | 0 syl = 0% | 5 syl (5-0) = 28% |
|  |  |  |  |  |  |  |  |  |  |
